# Role of epigenetics and the transcription factor Sp1 in the expression of the D prostanoid receptor 1 in human cartilage

**DOI:** 10.3389/fcell.2023.1256998

**Published:** 2023-11-30

**Authors:** Mehdi Najar, Sami G. Alsabri, Gadid G. Guedi, Makram Merimi, Frédéric Lavoie, Detlev Grabs, Jean-Pierre Pelletier, Johanne Martel-Pelletier, Mohamed Benderdour, Hassan Fahmi

**Affiliations:** ^1^ Osteoarthritis Research Unit, University of Montreal Hospital Research Center (CRCHUM), Montreal, QC, Canada; ^2^ Laboratory of Experimental Hematology, Institut Jules Bordet, Université Libre de Bruxelles (ULB), Brussels, Belgium; ^3^ Departement of Orthopedic Surgery, University of Montreal Hospital Center (CHUM), Montréal, QC, Canada; ^4^ Research Unit in Clinical and Functional Anatomy, Department of Anatomy, Université du Québec à Trois-Rivières, Trois-Rivières, QC, Canada; ^5^ Orthopedics Research Laboratory, Research Center, Hôpital du Sacré-Cœur de Montréal, Université de Montréal, Montréal, QC, Canada

**Keywords:** DP1, prostaglandin D2, chondrocyte, Sp1, epigenetics, DNA methylation, cartilage, osteoarthritis

## Abstract

D prostanoid receptor 1 (DP1), a prostaglandin D2 receptor, plays a central role in the modulation of inflammation and cartilage metabolism. We have previously shown that activation of DP1 signaling downregulated catabolic responses in cultured chondrocytes and was protective in mouse osteoarthritis (OA). However, the mechanisms underlying its transcriptional regulation in cartilage remained poorly understood. In the present study, we aimed to characterize the human DP1 promoter and the role of DNA methylation in DP1 expression in chondrocytes. In addition, we analyzed the expression level and methylation status of the DP1 gene promoter in normal and OA cartilage. Deletion and site-directed mutagenesis analyses identified a minimal promoter region (−250/−120) containing three binding sites for specificity protein 1 (Sp1). Binding of Sp1 to the DP1 promoter was confirmed using electrophoretic mobility shift assay (EMSA) and chromatin immunoprecipitation (ChIP) assays**.** Treatment with the Sp1 inhibitor mithramycin A reduced DP1 promoter activity and DP1 mRNA expression. Inhibition of DNA methylation by 5-Aza-2′-deoxycytidine upregulated DP1 expression, and *in vitro* methylation reduced the DP1 promoter activity. Neither the methylation status of the DP1 promoter nor the DP1 expression level were different between normal and OA cartilage. In conclusion, our results suggest that the transcription factor Sp1 and DNA methylation are important determinants of DP1 transcription regulation. They also suggest that the methylation status and expression level of DP1 are not altered in OA cartilage. These findings will improve our understanding of the regulatory mechanisms of DP1 transcription and may facilitate the development of intervention strategies involving DP1.

## Introduction

Osteoarthritis (OA) is the most common musculoskeletal disorder and a leading cause of long-term disability with significant socioeconomic impact. Clinical manifestations of OA may include pain, stiffness, and mobility impairment. Although several risk factors have been identified, the precise mechanisms underlying the initiation and/or progression of the disease are not fully understood. The main pathological hallmarks of OA include progressive cartilage degeneration, inflammation of the joint lining (synovium), and subchondral bone sclerosis (Martel-Pelletier et al., 2016; Wei and Bai, 2016; Hugle and Geurts, 2017). These changes have been largely attributed to the enhanced release of catabolic and inflammatory mediators (Martel-Pelletier et al., 2016; Wei and Bai, 2016; Hugle and Geurts, 2017). An improved understanding of the mechanisms involved in the initiation and progression of OA will be instrumental in developing new and more effective therapeutic strategies.

Epigenetic changes, particularly DNA methylation, play a prominent role in the regulation of gene expression. DNA methylation occurs at cytosine located within CpG dinucleotide and is generally associated with gene silencing (Edwards et al., 2017; Lyko, 2018). It is catalyzed by DNA methyltransferases (DNMTs) including, the maintenance methyltransferase DNMT1, and the *de novo* methyltransferases DNMT3a and DNMT3b (Edwards et al., 2017; Lyko, 2018). Several studies suggest that abnormal DNA methylation is implicated in the pathogenesis of OA. For instance, Shen et al. (2017) demonstrated that DNMT3B is downregulated in mouse and human OA cartilage. Importantly, cartilage-specific Dnmt3b deficiency accelerated, whereas Dnmt3b overexpression attenuated the development of OA in mice (Shen et al., 2017). Furthermore, DNA methylation at CpG sites was reported to regulate the expression of several genes involved in the pathogenesis of OA including the cartilage-degrading enzyme, matrix metalloproteinase-13 (MMP-13), and aggrecanase-1 (Cheung et al., 2009; Hashimoto et al., 2009), the pro-inflammatory cytokines, interleukin-1 (IL-1) and IL-8 (Hashimoto et al., 2009; Takahashi et al., 2015), and growth factors such as osteogenic protein-1 (OP-1), bone morphogenetic protein-7 (BMP-7) (Loeser et al., 2009), and growth differentiation factor 5 (GDF5) (Reynard et al., 2014).

Prostaglandin D_2_ (PGD_2_) is an essential endogenous lipid mediator generated from arachidonic acid. It exerts its biological functions via two G-protein-coupled receptors, the D prostanoid receptor (DP1) (Hirata et al., 1994), and the chemoattractant receptor-homologous molecule expressed on Th2 cells (CRTH2 or DP2) (Hirai et al., 2001). PGD_2_ plays important roles in numerous physiological and pathological processes including chemotaxis (Hirai et al., 2001), cell trafficking (Ahmed et al., 2011), bone metabolism (Yue et al., 2014) and cancer (Omori et al., 2018). PGD_2_ has also been reported to display anti-inflammatory properties via the DP1 receptors. For example, *in vitro* treatment with PGD_2_ or BW 245C, a selective DP1 agonist, inhibited several inflammatory responses, including tumor necrosis factor-α-induced migration of Langerhans cells (Angeli et al., 2001), and virus-induced inflammasome activation in Cd11b + cells (Vijay et al., 2017). PGD_2_/DP1 signaling was also shown to suppress the production of interferon γ by NK T cells (Torres et al., 2008), and histamine and leukotrienes by rings of the trachea (Safholm et al., 2021). Furthermore, activation of the PGD_2_/DP1 pathway decreased the expression of IL-12 and upregulated the expression the anti-inflammatory cytokine IL-10 in dendritic cells (Gosset et al., 2005). *In vivo* studies showed that administration of a DP1 activator was protective in several models of inflammatory conditions including collagen-induced arthritis (Maicas et al., 2012), colitis (Ajuebor et al., 2000), atopic dermatitis (Angeli et al., 2004), asthma (Hammad et al., 2007), and chronic allergic lung inflammation (Maehara et al., 2019). Further studies showed that DP1 deficiency exacerbated inflammation in zymosan-induced peritonitis and myocardial infraction (Kong et al., 2016), bleomycin-induced acute lung injury (van den Brule et al., 2014), ulcerative colitis (Li et al., 2017), and angiogenesis (Murata et al., 2008) in mice.

We have previously shown that DP1 is expressed in cartilage and that treatment with PGD_2_ or BW245C suppressed the expression of the cartilage-degrading enzymes MMP-1 and MMP-13 in cultured chondrocytes (Zayed et al., 2008a). Additionally, we demonstrated that administrating BW245 alleviated instability-induced OA in mice (Ouhaddi et al., 2017). We also found that deletion of DP1 accelerated and enhanced the severity of instability-induced and naturally occurring age-related OA in mice (Ouhaddi et al., 2017), suggesting that DP1 may constitute a promising therapeutic target in the treatment of OA.

Although DP1 has been shown to display protective properties in many inflammatory and degenerative diseases, including OA, little is known about its transcriptional regulation. In the present study, we have characterized the DP1 promoter in a human chondrocyte cell line. We also investigated the role of DNA methylation in its expression. In addition, we analyzed the expression level and the methylation status of the DP1 gene promoter in OA and control cartilage.

## Materials and methods

### Generation of luciferase reporter constructs

The human DP1 promoter region spanning nucleotides −1080 to +1 was amplified by PCR using genomic DNA from normal human articular chondrocytes as a template and forward and reverse PCR primers carrying KpnI and XhoI recognition sequences, respectively (Table 1). The amplified DNA fragment was digested with KpnI and XhoI (Thermo Fisher Scientific, Mississauga, ON, Canada), purified, and cloned into the pGL-3-basic luciferase reporter vector (Promega, Madison, WI).

**TABLE 1:** List of primers used for cloning, qRT-PCR, ChM and pyrosequencing, and probes used for EMSA.

Reaction	Primer sequence (5′to 3′)
Cloning into pGL3-basic	F: CATCAGGTACCAAAACACCCIT1CTATCAAA (Kpnl)
	R: AGTGAAGC.ICTGCGGAAGCTCGAGGGC (Xhol)
EMSA	
Spl -1	F: GAG​CGT​CCC​GCC​TCT​CAA​AGA​GG
	R: CCT​CTT​TGA​GAG​GCG​GAC​GCT​C
Spl -1 Mut	GAG​CGT​CCT​AAA​TCT​CAA​AGA​GG
	R: CCT​CTT​TGA​GAT​TTA​GGA​CGC​TC
Sp1-2a, 2b	F: GGG​AAC​ACC​CCG​CCG​CCC​TCG​GAG​CT
	R: AGC​TCC​GAG​GGC​GGC​GGG​GTG​TTC​CC
Spl -2a Mut	F: GGG​AAC​ACC​CTA​AAG​CCC​TCG​GAG​CT
	R: AGCMCGAGGGCTTTAGGOTGTTCCC
Spl -2b Mut	F: GGG​AAC​ACC​CCG​CTA​AAC​TCG​GAG​CT
	R: AGCTCCGAGTTTAGCOGGGTOTTCCC
Spl consensus	F: ATTCGATCGGGOCGOGGCGAGC
	R: GCT​CGC​CCC​GCC​CCG​ATC​GAA​T
5$ consensus Mut	F: ATT​CGA​TCG​GTT​CGG​GGC​GAG C
	R: GCT​CGC​CCC​GAA​CCG​ATC​GAA​T
ChIP (Sp I -a)	F: GGT​GGC​TGC​TGC​TTA​ATT​TC
	R: GGC​AGG​AAC​CTC​CTA​TCT​AAA​C
ChIP (Sp1-2a, 2b)	F: CTC​TCA​AAG​AGG​GGT​GTG​ACC
	R: AAGCTGCGCCACAGAAA
Pyrosequencing-1 (−250/-177)	F: TTGOTOTTGGGTGITTGGAATT
	R: ATATTCCCCACCACAAAAACCTCCrATCT
	S: GGTAGAGTTTTTTATTGGT7TOT
Pyrosequencing-1 (−153/-130)	F: GTTITTTAAAGAGGGGTGTGAT
	ATT​ACC​TTT​TTC​CAC​AAA​AAT​AAT​ATT​CT
	S: GAG​TTT​AGA​TAG​GAG​GTT​T
Real time RT-PCR	
DPI	F: ATA​GCC​GAA​AAG​GAG​CAC​AA
	R: CCT​GCA​AGC​TGG​GTT​TAG​AG
ACAN	F: GCC​TAT​CAG​GAC​AAG​GTC​TC
	R: ATG​ATG​GCA​CTG​TTC​TGC​AG
COL2	F: CAC​ACT​CAA​GTC​CCT​CAA​CAA
	R: AGT​AGT​CTC​CAC​TCT​TCC​ACT​C
ADAMTS5	F: GGC​ATC​ATT​CAT​GTG​ACA​C
	R: GCA​TCG​TAG​GTC​TGT​CCT​G
MMP-13	F: CTT​AGA​GGT​GAC​TGG​CAA​AC
	R: GCC​CAT​CAA​ATG​GGT​AGA​AG
HPRT	F: TGA​CCT​TGA​TTT​ATT​TTG​CAT​ACC
	R: CGA​GCA​AGA​CGT​TCA​GTC​CT
GAPDH	F: CAG​AAC​ATC​ATC​CCT​GCC​TCT
	R: GCT​TGA​CAA​AGT​GGT​CGT​TGA​G

Restriction sites included in the primers are underlined.

F, forward primer; R, reverse primer; S, sequencing primer.

DNA fragments corresponding to the DP1 promoter region from −550/+1, −374/+1, −250/+1, −170/+1 and −120/+1 with a KpnI restriction site at the 5′end and an XhoI restriction site at the 3′end, were synthesized (GeneWiz, Cambridge, MA) and subcloned into the pGL-3-basic vector. A set of mutant promoter constructs bearing mutation in each, two or all three putative Sp1 binding sites, was also generated by direct DNA synthesis (GeneWiz). The mutated nucleotide sequences are shown in Figure 2A. Similarly, a DNA fragment corresponding to the DP1 promoter region from −374 to +1 was synthesized (GeneWiz) and inserted at the *Kpn*I/*Xho*I sites of the pCpGL plasmid, a CpG-free luciferase reporter vector (Gebhard et al., 2010). All generated promoter constructs were verified by DNA sequencing.

### Transient transfection and reporter gene assays

Transient transfection experiments were performed using the Lipofectamine 3,000 transfection reagent (Thermo Fisher Scientific) following the manufacturer’s instructions. Briefly, the immortalized human chondrocytes, C28/I2 (kindly provided by Dr. Mary Goldring, Hospital for Special Surgery, NYC), were seeded at a 3.0 × 10^4^ cells/well density in 24-well plates, 1 day before transfection. Cells were transfected with 400 ng of the various DP1 reporter constructs and 10 ng of Renilla Luciferase Control Reporter plasmid (Promega, Madison, WI). In co-transfection assays, the expression vector for Sp1 (generously provided by Dr. Stephen Smale, UCLA) was used (10 ng/well). Forty hours post-transfection, cells were washed with PBS and lysed in 100 μL passive lysis buffer (Promega). Luciferase activity (20 μL of cell lysate) was measured using the Dual-Luciferase reporter assay system (Promega) following the manufacturer’s instructions. Each transfection was performed in triplicate, and each experiment was repeated at least three times.

### Electrophoretic mobility shift assay

Nuclear extracts were prepared and quantified as described previously (Cheng et al., 2004; Chabane et al., 2008). Oligonucleotides (Table 1) were synthesized and biotinylated on their 5′-end by Integrated DNA Technologies (Coralville, IA) and then annealed into double strands. Binding reactions were performed using a LightShift Chemiluminescent EMSA kit (Thermo Fisher Scientific) according to the manufacturer’s instructions. Briefly, 5 μg of nuclear extract was incubated with 20 fmol of the biotin-labeled probe in 1X binding buffer containing 50 ng/μL poly dI-dC, 2.5% glycerol, 5 mM MgCl2 and 0.05% NP40 in a final volume of 20 μL at room temperature for 20 min. In cold competition assays, 100-fold molar excess of cold wild-type or mutant oligonucleotide was used. In supershift assays, nuclear extracts were incubated with 1 μg of anti-Sp1 antibody (MilliporeSigma, Oakville, ON, Canada) for 15 min at room temperature before the addition of the probe. Binding complexes were resolved on a non-denaturing 6% polyacrylamide gel, transferred to a positively charged membrane (Invitrogen), and cross-linked with UV irradiation. Detection of biotin-labeled DNA was performed using the Chemiluminescent Nucleic Acid Detection Module (Thermo Fisher Scientific).

### Chromatin immunoprecipitation (ChIP) assay

ChIP assays were performed using the Magna ChIP Assay kit (MilliporeSigma) in accordance with the manufacturer’s protocol. Briefly, cells were treated with formaldehyde to cross-link DNA and associated proteins. Cell lysates were sonicated to shear chromatin to fragments between 200–500 bp. The chromatin samples were diluted 10-fold in ChIP dilution buffer, and an aliquot was saved as the input DNA. The DNA-protein complexes were immunoprecipitated with an anti-Sp1 antibody (MilliporeSigma). Normal rabbit IgG was used as the negative control. DNA fragments were eluted from each immunoprecipitation and subjected to PCR analysis. Fold enrichment was assessed using qRT-PCR and the 2^−ΔΔCT^ method.

The primers used are listed in Table 1.

### Cartilage samples and nucleic acid isolation

Macroscopically normal human knee cartilage was obtained at autopsy from 11 donors (5 females and 6 males) with no history of joint disease or trauma (mean age ±SD, 66 ± 10 years). The normality of the joint and integrity of cartilage were confirmed macroscopically at the time of sample collection. OA cartilage was obtained from 28 OA patients (17 females and 11 males) who underwent total knee replacement surgery (mean age ±SD, 70 ± 9 years). All patients had a Kellgren-Lawrence grade ≥3. Demographic characteristics of the donors are shown in Table 2. Informed consent was obtained from each donor, and the local ethics committee approved the study. In normal donors, cartilage samples were collected from the medial femoral condyle. In OA donors, cartilage was also collected from the medial femoral condyle, but only from macroscopically unaffected regions. Tissues were taken from the three (superficial, intermediate, and deep) layers of articular cartilage.

**TABLE 2:** Characteristics of cartilage donors.

	Controls (*n* = 11)	OA patients (*n* = 28)	*p*-value
Age (years)	66 ± 10	70 ± 9	0.21
Sex, female n (%)	5 (45%)	17 (60%)	0.74
Height (cm)	169.6 ± 10.6	162.5 ± 10.9	0.07
Weight (kg)	79.8 ± 18.2	86.6 ± 21	0.30
BMI (kg/m2)	27.7 ± 5.7	32.7 ± 8.8	0.08

OA, osteoarthritis; BMI, body mass index Data are presented as mean ± S. D, or as n (%) and were analyzed using the Student’s t-test or chi-squared test, as appropriate.

Cartilage samples were cut into small fragments, divided into portions for DNA and RNA extraction, flash-frozen in liquid nitrogen, and stored at −80°C until use. Samples were ground in liquid nitrogen and DNA, or RNA was isolated from each sample, using the DNeasy plant maxi kit or the RNAeasy Mini kits (Qiagen, Toronto, ON, Canada), respectively.

### Real-time quantitative polymerase chain reaction

RNA was reverse transcribed and amplified using a QuantiTect RT-PCR kit (Qiagen) on a Rotor-Gene 3,000 real-time PCR system (Corbett Research), according to the manufacturer’s protocol. All values were normalized to the mean CT value of two reference genes, glyceraldehyde 3-phosphate dehydrogenase (GAPDH) and hypoxanthine-guanine phosphoribosyltransferase 1 (HPRT1). Data were expressed as relative gene expression using the 2^−ΔΔCT^ method or as fold change using the 2^−ΔΔCT^ method, as previously described (Livak and Schmittgen, 2001; Zayed et al., 2008a; Ouhaddi et al., 2017). Each PCR was performed in triplicate from 3 independent experiments. Primers are listed in Table 1.

### Pyrosequencing

Genomic DNA (500 ng) isolated directly from knee cartilage and from cultured chondrocytes was bisulfite converted using an EpiTect Bisulfite Kit (Qiagen) according to the manufacturer’s instructions. PCR was performed using the TaKaRa EpiTaq HS kit (Cedarlane, Burlington, ON, Canada) following the supplier’s instructions. The size and purity of the PCR products were confirmed by agarose gel electrophoresis. Pyrosequencing was carried out using a PyroMark Q24 system/instrument (Qiagen). PCR primers and sequencing primers (Table 1) were designed using PyroMark Assay Design 2.0 software (Qiagen). The methylation percentage at each CpG site of the DP1 promoter was determined using the PyroMark Q24 software version 2.0.6.

### Chondrocyte culture and 5-Aza-dC treatment

Primary human articular chondrocytes were released from cartilage by sequential enzymatic digestion with 2 mg/mL pronase (MilliporeSigma) for 1 h and 1 mg/mL collagenase type II (Thermo Fisher Scientific) for 6 h at 37°C. Cells were seeded at 1.2 × 10^4^ cells/cm^2^ in 25 cm^2^ flaks in DMEM supplemented with 10% (heat-inactivated) FCS and then cultured at 37°C for 72 h. Cells were treated either with the vehicle or with 5 μM 5-Aza-2′-deoxycytidine (5-Aza-dC) for 5 weeks, adding freshly prepared drug (5-Aza-dc) twice weekly. Finally, cells were washed with ice-cold PBS, and total RNA and DNA were isolated. Gene expression and DNA methylation were evaluated as described above.

### *In vitro* DNA methylation

The pCpGL-DP1-374/+1 luciferase reporter construct was methylated using the CpG methyltransferase, M. SssI (New England Biolabs, Whitby, ON, Canada). Briefly, plasmid DNA was incubated with 4 units of methyltransferase per μg DNA in the presence of 160 μM S-adenosylmethionine (SAM) for 4 h at 37°C. After the first 2 hours, the methylation reaction was supplemented with a further dose of 160 μM SAM. Mock methylation reactions in which SssI methyltransferase was omitted were performed in parallel. Successful methylation was verified by digestion with the restriction enzyme Hpa II (New England Biolabs). Methylated and mock-methylated plasmids were purified using a QIAquick PCR Purification Kit (Qiagen).

### Statistical analysis

Data from transfection assays and real-time PCR analyses in cultured cells were assessed by one-way ANOVA, followed by either the Tukey or the Dunnett’s *post hoc* test for comparison of multiple groups. Comparison between two groups was performed using the Student’s t-test or chi-squared test, where appropriate. Comparison of the level of CpG methylation between groups was assessed using the Student’s t-test with Bonferroni correction for multiple testing. Relative DP1 expression in control and OA cartilage were compared using the Mann-Whitney *U* test. *p*-value <0.05 was considered significant. All analyses were performed using Prism 9.0 (GraphPad).

## Results

### Delineation of the human DP1 promoter

**FIGURE 1:**
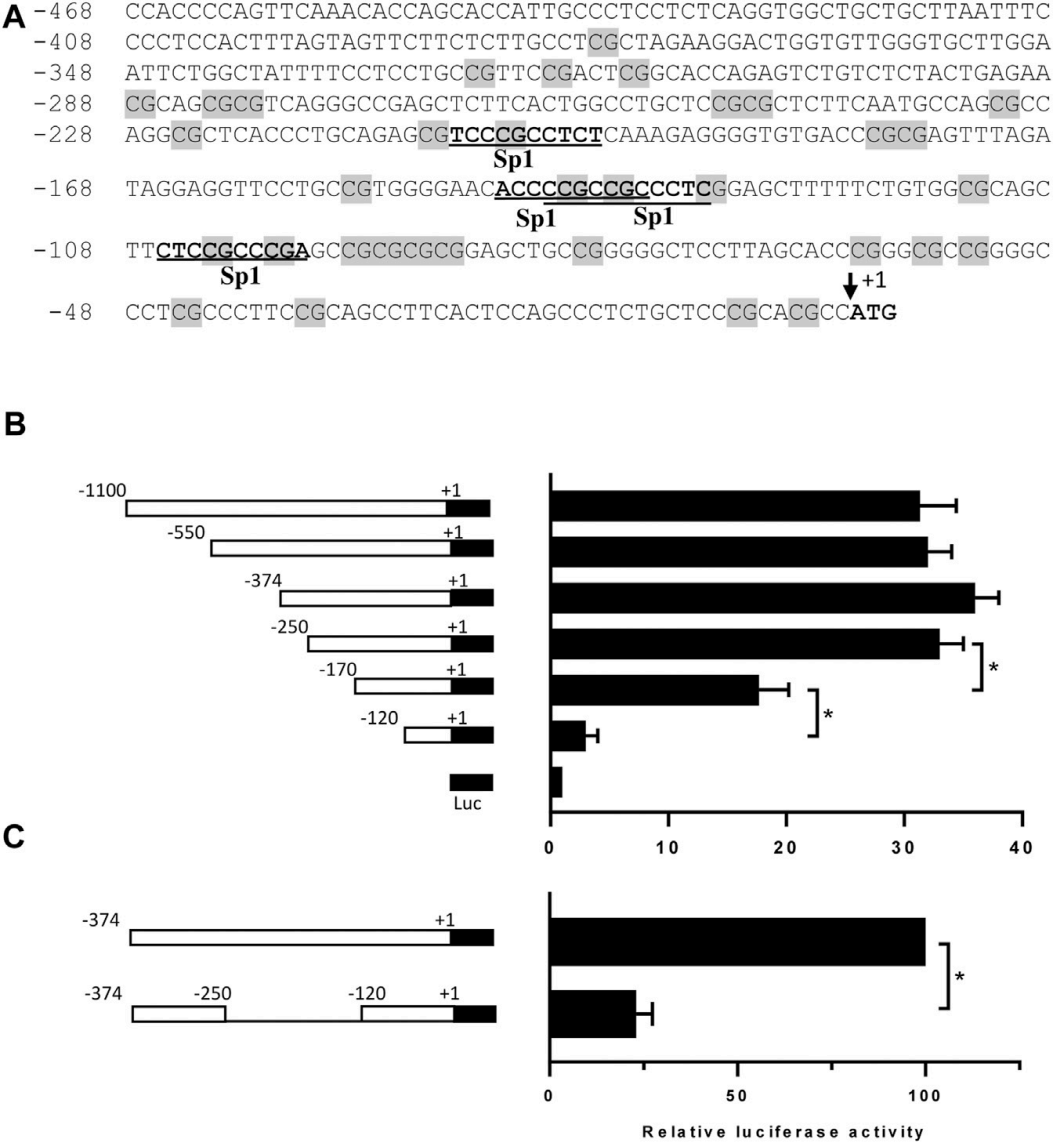
Nucleotide sequence and functional analysis of the human DP1 promoter in C28/I2 cells **(A)** Nucleotide sequence and putative regulatory elements within bp −468/+1 of the 5′-flanking region of the human DP1 gene. The translation initiation codon, ATG, is indicated as +1. CpG dinucleotides are shaded in grey on the sequence. The potential binding sites for the transcription factor Sp1 are underlined. **(B)** C28/I2 chondrocyte cells were co-transfected with different DP1 promoter constructs, as indicated on the left panel, and a Renilla reporter plasmid. After 40 h, luciferase activity was determined, normalized to Renilla activity, and expressed as fold increases over that of the pGL3-basic vector, which was assigned a value of 1. Data are mean ± SD of three experiments performed in triplicate and were analyzed using one-way ANOVA followed by the Tukey test. *p < 0.05. **(C)** The region −250/-120 was deleted in the construction −374/+1-Luc, and each construct was co-transfected with a Renilla reporter plasmid in C28/I2 cells. Luciferase activity was determined, normalized to Renilla, and was expressed as percent activity of the −374/+1-Luc WT construct, which was set at 100%. Data are mean ± SD of three experiments performed in triplicate and were analyzed using the Student’s t-test. *p < 0.05.

To characterize the transcriptional mechanisms underlying DP1 expression, a 1080-bp fragment containing the 5′-flanking region upstream of the translation start site (ATG) of the human DP1 gene (GenBank accession number NG_012118.1) was cloned into the pGL3-basic luciferase vector. As shown in Figure 1A, this region lacks a TATA box and contains GC-rich sequences. DNA sequence analysis revealed four putative Sp-1 binding motifs between positions −206 and −97 (Figure 1A).

To delineate the minimal DP1 promoter region required for basal DP1 promoter activity, a series of luciferase report constructs harboring various lengths of the DP1 promoter were generated. These constructs were then transiently transfected into the C28/I2 chondrocyte cell line, which expresses DP1 constitutively. As shown in Figure 1B, the fragments −1080/+1, −550/+1, −374/+1 and −250/+1 had virtually similar promoter activity, showing an increase in luciferase activity of 31-, 32-, 36- and 33-fold, respectively when compared to the empty pGL3-Basic vector. 5’ deletion of the −250/+1 construct from −250 to −170, and −120, gradually and drastically reduced luciferase activity, suggesting that the region between −250 and −120 contains essential element(s) for transcriptional activity of the human DP1 promoter. The observation that deletion of this region while maintaining the upstream and downstream portions, resulted in an 80% decrease in the promoter activity (Figure 1C) further supports that the primary promoter regulatory elements are located between −250 and −120 bp.

### Sp1 activates the DP1 promoter

**FIGURE 2:**
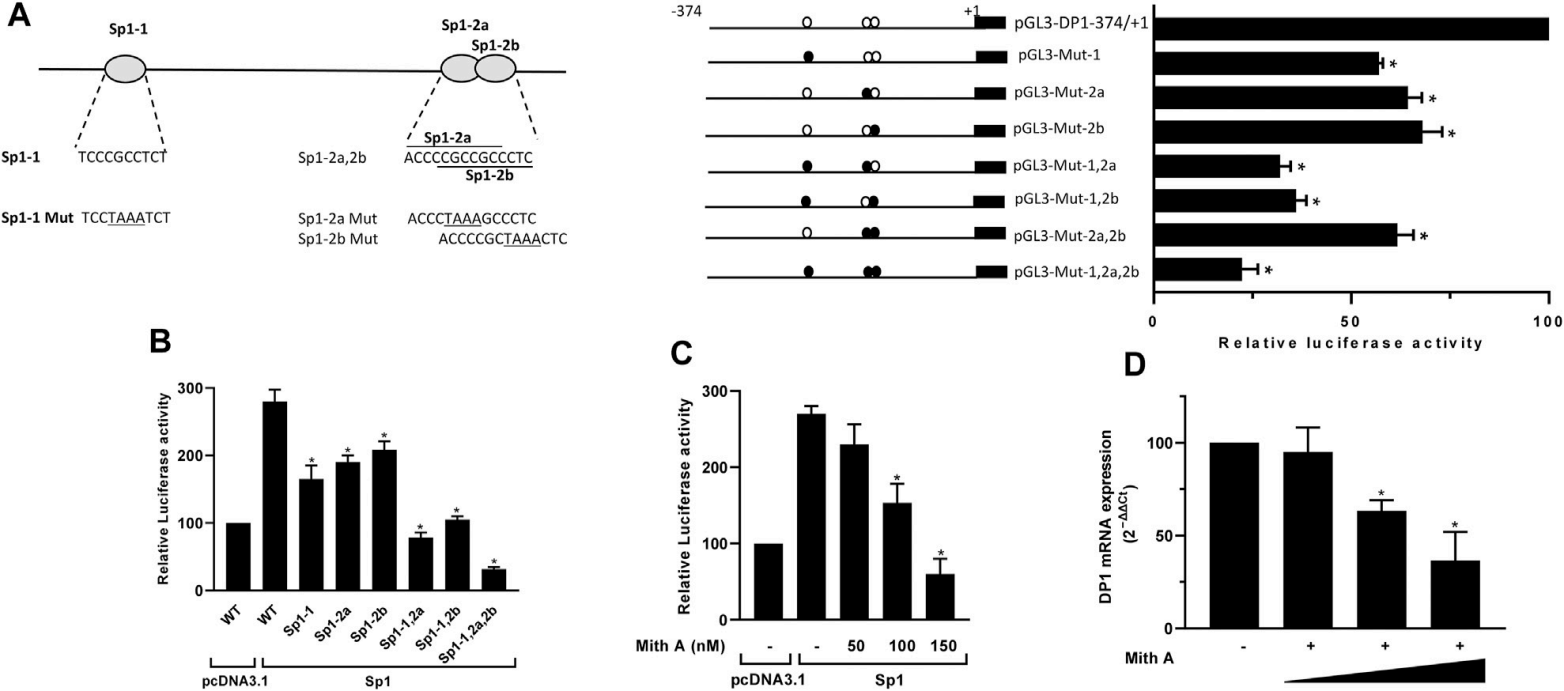
Sp1 regulates DP1 promoter activity and gene expression **(A)** Effect of mutating Sp1 binding sites on DP1 promoter activity. Left panel: nucleotide sequences of wild and mutant of three putative Sp1 binding sites (Sp1-1, Sp1-2a and Sp1-2b) in the DP1 promoter. Mutated bases are underlined. Right panel: DP1 promoter (−374/+1) construct or reporter constructs carrying single, double, or triple mutations in the three predicted Sp1 binding sites were transfected into C28/I2 cells. After 40 h, luciferase activity was determined and normalized to Renilla activity. The activity of each construct is expressed as a percentage of that of the WT promoter (−374/+1) construct, which was set at 100*%*. **(B)** Overexpression of Sp1 enhances DP1 promoter activity. C28/I2 cells were co-transfected with DP1 promoter (−374/+1) construct or mutated luciferase reporter vectors and an Sp1 expression vector or the pcDNA3.1 empty vector (control) along with the *Renilla* luciferase plasmid. Reporter activity is expressed as the percent activity of the WT promoter construct (−374/+1) in pcDNA3.1-transfected cells, which was set at 100*%*. **(C)** Effect of Mith on basal and Sp1-induced DP1 promoter activity. C28/I2 cells were co-transfected with the DP1 promoter (−374/+1) construct and an Sp1 expression vector or the pcDNA3.1 empty vector (control) along with the *Renilla* luciferase plasmid. Eighteen hours post-transfection, cells were treated with vehicle or with increasing Mith concentration (50, 100 and 150 nM) for an additional 24 h. The reporter activity is expressed as the percent of that of the WT promoter (−374/+1) construct in pcDNA3.1-transfected and vehicle-treated cells, which was set at 100*%*. **(D)** Effect of Mith on DP1 mRNA expression. Cells were treated with vehicle or with increasing concentrations of Mith (50, 100 and 150 nM) for 24 h. Total RNA was isolated and reverse-transcribed into cDNA, and the DP1 level was quantified using real-time qPCR. Results are expressed as percentage of control, considering 100% as the value in vehicle-treated cells. Data are presented as the mean ± S. D from 3 independent experiments performed in triplicate and were analyzed using one-way ANOVA followed by the Dunnett’s test. *p < 0.05.

The −250/-120 fragment of the DP1 promoter includes three putative binding sites for Sp1, two of which overlap, and are defined as Sp1-1 (−206 to −196), Sp1-2a (−142 to −132) and Sp1-2b (−139 to −129) (Figure 2A). To determine whether the three putative Sp1 binding sites are essential for the transcriptional activation of DP1, we conducted transient transfection experiments with the WT DP1 promoter (−374/+1) construct or reporter constructs carrying a mutation in each, two, or all three Sp1 sites. As shown in Figure 2A, the single mutant constructs Sp1-1, Sp1-2a, and Sp1-2b displayed 43%, 36%, and 32% reduced activity, respectively, compared with the WT construct. The double mutants Sp1-1,2a (Sp1-1 and Sp1-2a mutated) and Sp1-1.2b (Sp1-1 and Sp1-2b mutated) displayed reduced activity by 68% and 64%, respectively, exhibiting an additive effect. The double mutant Sp1-2a, 2b (Sp1-2a and Sp1-2b mutated) displayed 38% reduced transcriptional activity. This value is virtually similar to that observed with individual mutants indicating, as expected with overlapping sites, that the effects of both mutations were not additive. Mutation of all sites reduced the activity by ∼80% (Figure 2A), suggesting that the three sites are essential for the regulation of the DP1 promoter.

Next, we tested the effect of Sp1 overexpression on the DP1 promoter activity. Cells were co-transfected with the WT DP1 promoter (−374/+1) construct, or the mutant constructs (Sp1-1, Sp1-2a, Sp1-2b, and Sp1-1,2a, 2b), and an expression vector encoding Sp1. As shown in Figure 2B, overexpression of Sp1 activated the wild-type DP1 promoter. The response of the single mutant constructs Sp1-1, Sp1-2a and Sp-1.2b to Sp1 overexpression was reduced by 41%, 32% and 26%, respectively, compared with the WT construct. The double mutant constructs Sp1-1,2a and Sp1-1.2b also displayed reduced response to Sp1 overexpression by 72% and 61%, respectively. The triple mutant Sp1-1,2a, 2b did not respond at all to Sp1 overexpression (Figure 2B).

To further assess the role of Sp1 in DP1 expression, cells were transfected with the DP1 promoter (−374/+1) construct and treated with increasing concentrations of mithramycin A (Mith), an Sp1 inhibitor. As shown in Figure 2C, treatment with Mith suppressed transactivation of the DP1 promoter by Sp1, in a dose dependent manner. Additionally, Mith treatment dose-dependently downregulated DP1 mRNA expression in primary OA chondrocytes (Figure 2D). These findings suggest that Sp1 is important for transcriptional regulation of DP1.

### Sp1 specifically binds to the DP1 promoter

**FIGURE 3:**
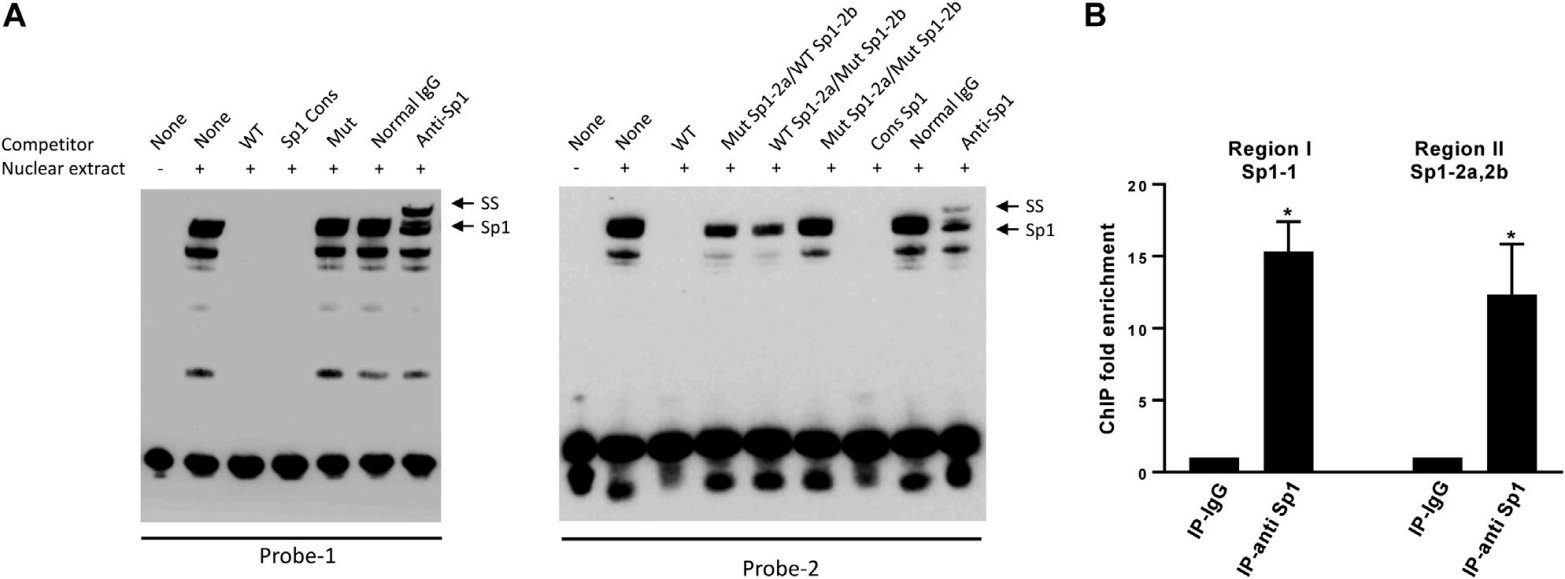
Sp1 binds to the DP1 promoter **(A)** EMSA analysis of Sp1 binding to the DP1 promoter sequence. Nuclear extracts from OA chondrocytes were incubated with a biotinylated oligonucleotide probe encompassing the Sp1-1 site (probe-1, left panel) or a biotinylated oligonucleotide probe encompassing both Sp1-2a and Sp1-2b sites (probe-2, right panel) and subjected to an EMSA. Competition assays were performed in the presence of 100-fold molar excess of the indicated unlabeled oligonucleotides, or with 1 μg of Sp1 antibody. Positions of the Sp1 and Sp1 supershifted band (*SS*) are indicated by arrows. A representative result of four independent experiments is shown. **(B)** ChIP analysis for Sp1 binding to the DP1 promoter in chondrocytes. Cross-linked chromatin from human OA chondrocytes was immunoprecipitated with normal rabbit IgG (IgG), or an anti-Sp1 antibody. Immunoprecipitated DNA was amplified and analyzed by real-time qPCR using primer sets amplifying the promoter regions encompassing the Sp1-1 site (region I) or the promoter region encompassing both Sp1-2a and Sp1 2b (region II). Fold enrichment of each region was calculated from qPCR data. Results are expressed as mean ± SD of three experiments performed in triplicate and were analyzed using the Student’s t-test. *p < 0.05.

To determine whether Sp1 binds to the predicted binding sites at the DP1 promoter, we performed EMSA analysis using the biotin-labeled oligonucleotides encompassing the putative binding sites, and nuclear extracts from OA chondrocytes. As shown in Figure 3A, DNA-protein complexes were formed with the probe encompassing Sp1-1 (left panel, lane 2). These complexes disappeared in the presence of a 100-fold molar excess of unlabeled wild-type probe (lane 3) and a similar excess of a Sp1 consensus oligonucleotide (lane 4). Incubation with the unlabeled mutant oligonucleotide probe has no effect on the formation of these complexes (lane 5). The biotin-labelled probe encompassing Sp1-2a and Sp1-2b also formed DNA-protein complexes when incubated with nuclear extracts from chondrocytes (right panel, lane 2). Formation of these complexes was prevented in the presence of a 100-fold molar excess of an unlabeled WT probe (lane 3) or a Sp1 consensus oligonucleotide (lane 7). Since Sp1-2a and Sp1-2b overlap, we conducted competition experiments using oligonucleotides in which each Sp1 motif was individually mutated and one in which both motifs were simultaneously mutated. Competition with these oligonucleotides revealed that oligonucleotide mutated for Sp1-2a or Sp1-2b partially abolished the formation of the DNA-protein complex (lanes 4 and 5), whereas the double mutant oligonucleotide displayed no effect (lane 6). Supershift analyses revealed that DNA-complexes formed with both probes were shifted by anti-Sp1 antibody. No supershift was observed with the control IgG (Figure 3A).

We also performed ChIP assays to determine whether Sp1 binds to the DP1 promoter *in vivo*. Cross-linked chromatin from OA chondrocytes was immunoprecipitated with an anti-Sp1 antibody or a normal rabbit IgG (negative control). DNA isolated from the immunoprecipitates was analyzed by PCR using primers sets specific to each of the two regions containing the putative Sp1 binding sites. ChIP-qPCR assays showed that the promoter region encompassing the Sp1-1 site and the region encompassing both Sp1-2a and Sp1-2b sites were enriched in immunocomplexes using an anti-SP1 antibody by 15- and 12-fold, respectively, while no enrichment was detected in those using normal IgG. These results confirmed that Sp1 can bind to the endogenous DP1 promoter.

### Inhibition of DNA methylation with 5-Aza-dc upregulates DP1 expression in articular chondrocytes

**FIGURE 4:**
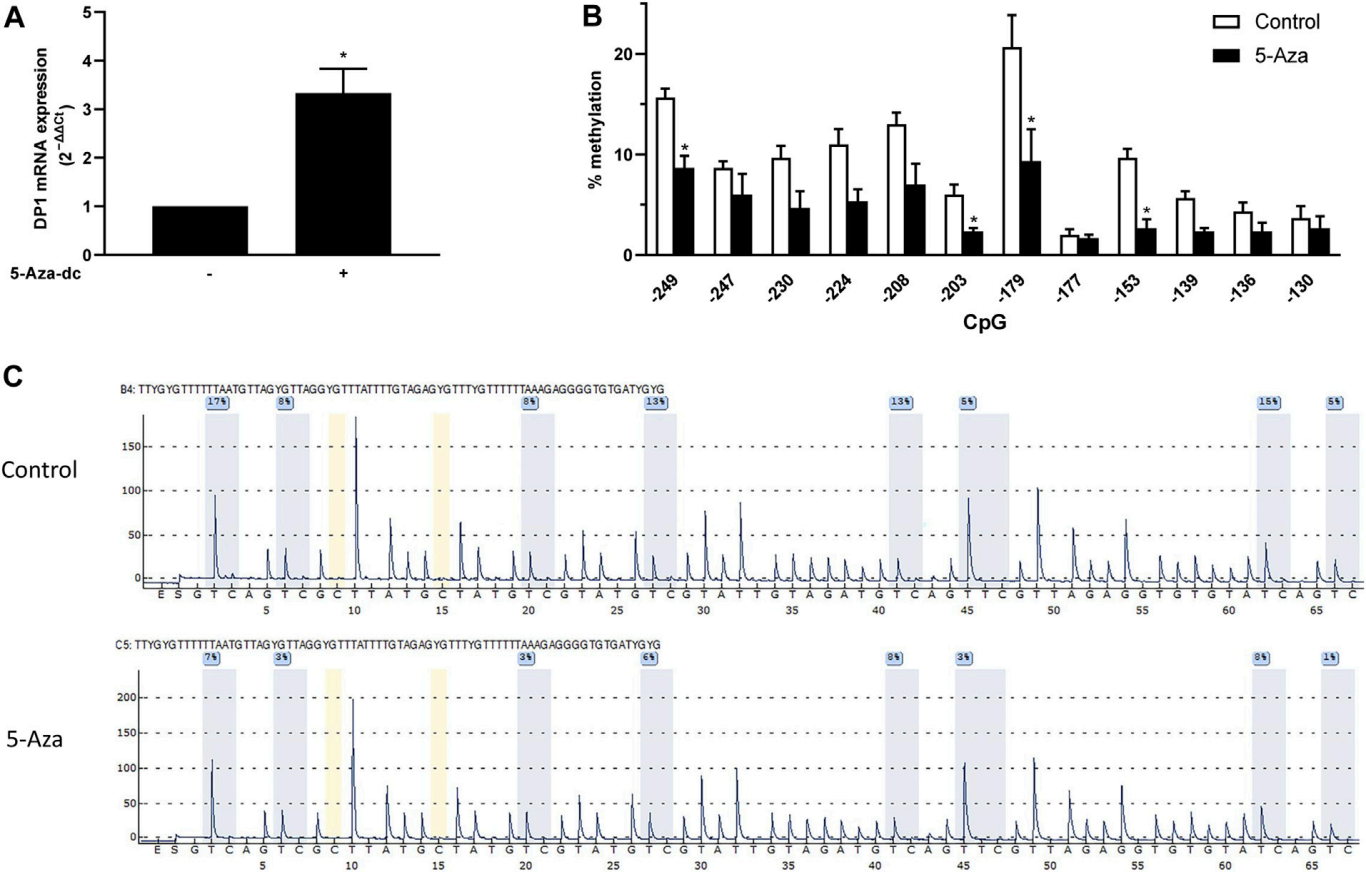
CpG demethylation with 5-Aza-dC enhanced DP1 levels in human chondrocytes OA chondrocytes were treated either with vehicle or with 5 μM 5-Aza-dC for 5 weeks. Freshly prepared 5-Aza-dc was added twice weekly. DNA and RNA were extracted. **(A)** Total RNA was reverse-transcribed into cDNA, and DP1 mRNA levels were determined using real-time qPCR. All experiments were performed in triplicate, and negative controls without template RNA were included in each experiment. Results are expressed as fold changes, considering 1 as the value of vehicle-treated cells. Data are presented as the mean ± S. D from 3 independent experiments performed in triplicate and were analyzed using the Student’s t-test. *p < 0.05. **(B)** The level of methylation of the indicated CpG in the DP1 promoter was determined using bisulfite pyrosequencing. Values are expressed as mean ± SD. Data were analyzed using the Student’s t-test with Bonferroni correction for multiple testing)**. (C)** Representative pyrograms of the DP1 promoter region from −249 to −177 in control and OA cartilage. The sequence at the top of the pyrogram represents the sequence to be analyzed. The *y*-axis represents the signal intensity in arbitrary units, while the *x*-axis shows the dispensation order; E, enzyme mix; S, substrate; A, G, C, and T, nucleotide. Shaded bars highlight the analyzed CpG sites. Values in blue boxes are the percentages of methylation of each CpG after bisulfite conversion.

As mentioned above, the 5′-flanking region of the DP1 gene contains a CpG-rich area, suggesting that methylation of CpG dinucleotides might be involved in regulating DP1 expression. To test this possibility, we investigated the effect of the demethylating agent 5-Aza-dc on DP1 mRNA levels in human primary OA articular chondrocytes. DP1 mRNA levels in 5-Aza-dc treated cells were increased by 3.3-fold compared to control cultures (Figure 4A). Treatment with 5-Aza-dc had no effect on the mRNA and protein levels of Sp1 (data not shown), suggesting that the upregulation of DP1 expression by 5-Aza-dc was independent of an increase in Sp1 levels.

To evaluate whether the enhanced levels of DP1 mRNA in 5-Aza-dc-treated cells was associated with DNA demethylation, the methylation status of the DP1 promoter was quantified by bisulfite pyrosequencing. Methylation analysis performed with the corresponding samples indicates that the increased expression of DP1 was accompanied by reduced methylation levels of the CpG motifs contained in the minimal promoter region (−250 to −120 bp) (Figure 4B). Representative pyrograms of the 8 CpG motifs in region −249 to −177 are shown in Figure 4C.

These results suggest that demethylation of the DP1 promoter may contribute to the regulation of DP1 expression in chondrocytes.

### Effect of DNA methylation on the DP1 promoter activity and Sp1 binding to the DP1 gene promoter

**FIGURE 5:**
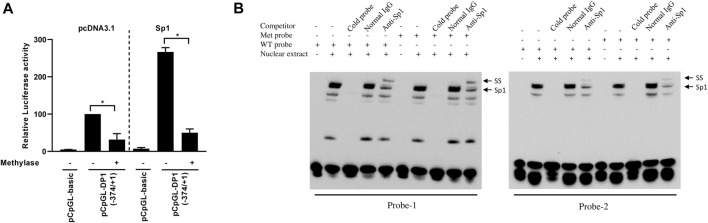
Effect of DNA methylation on DP1 transcriptional activity and Sp1 binding to the DP1 gene promoter **(A)** Effect of methylation on DP1 transcriptional activity. The DP1 promoter (pCpGL-374/+1) construct was *in vitro* methylated (Met), or mock-methylated (mock), with *Sss*I methylase and transfected into C28/I2 cells with the pcDNA3.1 or the Sp1 expression vector. After 40 h, luciferase activity was determined and normalized to Renilla activity. Results are expressed as percent activity of the mock-methylated pCpGL-374/+1 construct in pcDNA3.1-cotrasfected cells, which was set at 100%. Data are presented as the mean ± S. D from 3 independent experiments performed in triplicate and were analyzed using the Student’s t-test. *p < 0.05. **(B)** Effect of DNA methylation on the binding of Sp1 to the DP1 promoter. Nuclear extracts from OA chondrocytes were incubated with a biotinylated oligonucleotide probe encompassing the Sp1-1 site (probe-1, left panel) or a biotinylated oligonucleotide probe encompassing both Sp1-2a and Sp1-2b sites (probe-2, right panel), containing unmethylated (WT) or methylated CpG (Met) within the Sp1 binding sites and subjected to an EMSA analysis. Competition assays were performed in the presence of 100-fold molar excess of unlabeled probes. Supershift analysis was performed using an anti-SP1 antibody. Positions of the Sp1 and Sp1 supershifted bands (*SS*) are indicated by arrows. Representative results of four independent experiments are shown.

To determine whether CpG methylation of the DP1 promoter alters its transcriptional activity, the 374-bp (−374/+1) promoter fragment was cloned into the CpG-free luciferase vector pCpGL (pCpGL-374/+1). The reporter construct was methylated *in vitro* using CpG methyltransferase (M.SssI). Successful methylation was confirmed by enzymatic digestion with HpaII, a methylation-specific restriction enzyme. C28/I2 cells were transfected with the methylated or unmethylated (control) construct and luciferase activity was quantified. The results showed that methylation of the DP1 promoter dramatically decreased basal DP1 promoter activity when compared with the unmethylated one (Figure 5A). Sp1-induced activation of the DP1 promoter was also suppressed by DNA methylation (Figure 5A). These results demonstrate that DNA methylation downregulates the DP1 promoter activity.

To investigate whether DNA methylation affects the binding of Sp1 to its binding sites at the DP1 promoter, we performed EMSAs using oligonucleotide probes in which the CpG sites within the Sp1 binding sites were unmethylated or methylated. As reported above, DNA–protein complexes formed when nuclear extracts from chondrocytes were incubated with unmethylated probes (Figure 5B, lane 2 in the left and right panels). Similar EMSA band patterns and intensities were observed in the presence of methylated probes (Figure 5B *,* lane 7 in the left and right panels). The addition of a 100-fold molar excess of unlabeled probe prevented the formation of the DNA-protein complex (lanes 3 and 8). Incubation with an anti-Sp1 antibody supershifted the protein-DNA complex (lanes 5 and 10) consistent with Sp1-specific binding to both probes. These results suggest that CpG methylation at the Sp1 binding site did not affect the binding of Sp1 to the DP1 promoter.

### DP1 methylation status and expression level in control and OA cartilage

**FIGURE 6:**
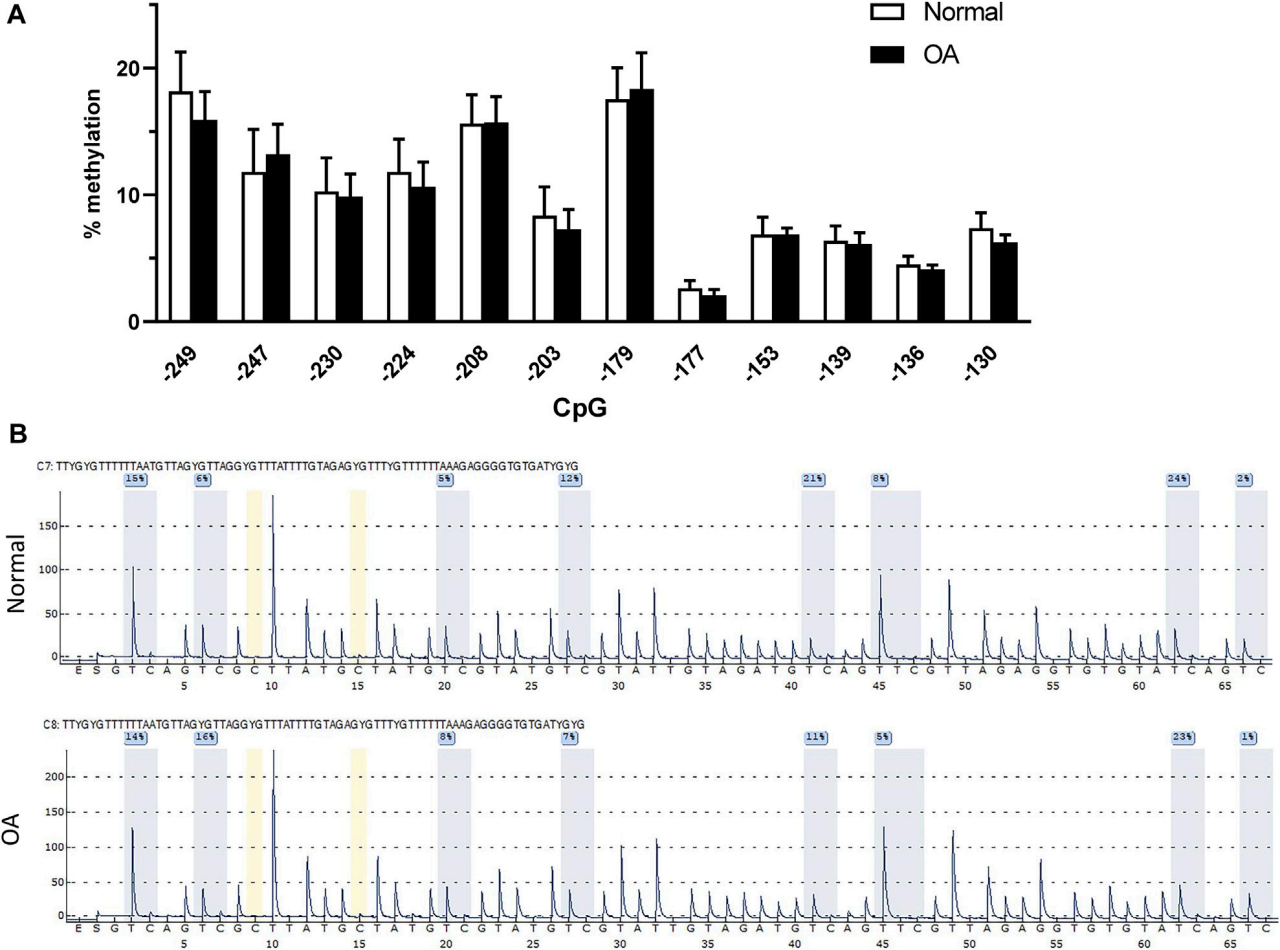
DP1 methylation and expression in control and OA cartilage **(A)**. Methylation status of the core DP1 promoter. Percentage DNA methylation of the indicated CpG sites on the proximal DP1 promoter in 11 normal (white bars) and 14 OA (black bars) cartilage, as determined by bisulfite pyrosequencing. Data were analyzed using the Student’s t-test with Bonferroni correction for multiple testing. **(B)** Representative pyrograms of the DP1 promoter region from −249 to −177 in control and OA cartilage. The sequence at the top of the pyrogram represents the sequence to be analyzed. The *y*-axis represents the signal intensity in arbitrary units, while the *x*-axis shows the dispensation order; E, enzyme mix; S, substrate; A, G, C, and T, nucleotide. Shaded bars highlight the analyzed CpG sites. Values in blue boxes are the percentages of methylation of each CpG after bisulfite conversion.

DNA methylation was reported to modulate the expression of many genes involved in the pathophysiology of OA (Cheung et al., 2009; Hashimoto et al., 2009; Loeser et al., 2009; Reynard et al., 2014; Takahashi et al., 2015). We therefore evaluated the methylation status of the −250/−120 bp proximal promoter region of DP1 in genomic DNA isolated directly from normal (*n* = 11) and OA (*n* = 14) cartilage. Pyrosequencing analysis revealed no difference in the methylation level at any CpG site analyzed between OA and normal cartilage (Figure 6A). Typical pyrosequencing results of the DP1 promoter region from −249 to −177 are shown in Figure 6B.

**FIGURE 7:**
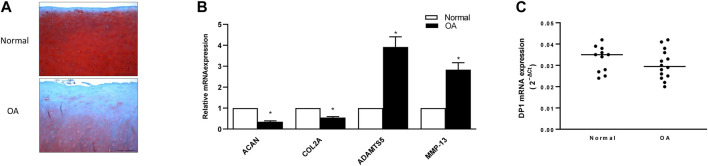
Expression of DP1 in control and OA cartilage **(A)** Representative images of Safranin-O stained normal and OA cartilage. Scale bars = 50 µm. **(B)** Relative mRNA expression of COL2, ACAN, MMP-13 and ADAMTS5 in normal and OA cartilage (*n* = 5). Results are expressed as fold changes, considering 1 as the value of control and are the mean ± S. D Data were analyzed using the Student’s t-test. *p < 0.05. **(C)** Relative mRNA expression of DP1 in normal (*n* = 11) and OA (*n* = 14) cartilage. Bars represent the median. Data were analyzed using the Mann-Whitney *U* test.

We also characterized the expression levels of DP1 in normal and OA cartilage. First, we compared histologic features in normal and OA cartilage. Safranin O staining showed that glycosaminoglycan (GAG) content was markedly reduced in OA cartilage than in normal cartilage. Moreover, OA cartilage displayed surface irregularities and small fibrillations, while normal cartilage appeared intact (Figure 7A). Next, we evaluated the expression level of OA related gene markers in normal and OA cartilage. As previously reported (Yang et al., 2016; Zhou et al., 2020), the mRNA expression of ACAN and COL2 were significantly decreased in OA cartilage compared with normal cartilage, whereas MMP-13 and ADAMTS5 mRNA expression were elevated in OA tissues (Figure 7B). As shown in Figure 7C, real-time qPCR analysis revealed no difference in DP1 mRNA expression level between OA and normal cartilage. These data suggest that the methylation status and the expression level of DP1 are not dysregulated in OA cartilage.

## Discussion

Accumulating evidence indicates that PGD_2_/DP1 signaling has anti-inflammatory effects and is protective in numerous models of inflammatory and degenerative conditions (Ajuebor et al., 2000; Angeli et al., 2004; Hammad et al., 2007; Murata et al., 2008; Maicas et al., 2012; van den Brule et al., 2014; Kong et al., 2016; Li et al., 2017; Maehara et al., 2019). We have previously shown that DP1 activation inhibits catabolic responses in cultured human chondrocytes (Zayed et al., 2008a) and displays protective properties in mouse OA (Ouhaddi et al., 2017). However, little is known about the mechanisms underlying DP1 transcriptional regulation. Here, we have characterized the human DP1 promoter and the role of DNA methylation in its expression in human chondrocytes. Additionally, we investigated the methylation status of the core promoter region of the DP1 gene and its expression levels in normal and OA cartilage.

Analysis of the human DP1 gene promoter activity using a series of luciferase report constructs and the human chondrocyte cell line C28/I2 showed that the minimal promoter is located between positions −250 to −120 bp upstream of the ATG. Computational analysis of this promoter region indicated the presence of three Sp1 binding sites. Sp1 has been considered a ubiquitous transcription factor involved in transcriptional activation of many housekeeping and tissue-specific genes (O'Connor et al., 2016). Interestingly, Sp1 was reported to regulate the expression of several chondrocyte-specific genes such as SOX9 (Piera-Velazquez et al., 2007), type II collagen (Ghayor et al., 2001), and type X collagen (Long et al., 1998). Sp1 was also shown to contribute to the transcriptional regulation of many prostaglandin receptors including, EP4 (Chien and Macgregor, 2003), FP (Zaragoza et al., 2004), TX (D'Angelo et al., 1996; Gannon and Kinsella, 2008), ALX/FPR2 (Simiele et al., 2012) and IP (Turner and Kinsella, 2009). Mutagenesis analyses revealed that the three Sp1 binding sites are essential for basal DP1 promoter activity. Mutation of the three sites did not completely abrogate basal DP1 promoter activity suggesting that additional factors may contribute to the regulation of the DP1 promoter. We have also demonstrated that co-transfection with an Sp1 expression vector upregulated the transcriptional activity of the DP1 promoter (−374/+1 construct). Additionally, our EMSA and ChIP analyses revealed that Sp1 could bind to the DP1 promoter. Moreover, treatment with Mith, an inhibitor of Sp1 binding, significantly reduced basal and Sp1-mediated promoter activity as well as the expression level of DP1 mRNA. Together, these data indicate that Sp1 is a key regulator of DP1 transcription.

Epigenetic mechanisms such as DNA methylation play key roles in regulating gene transcription (Edwards et al., 2017; Lyko, 2018). The coincidence of the minimal promoter sequence and a CpG rich region in the DP1 promoter suggests that DNA methylation could contribute to the regulation of DP1 expression. Indeed, we found that treatment with the demethylating agent 5-Aza-dC increased the levels of DP1 mRNA in human OA chondrocytes, and the observed changes were associated with reduced methylation levels of the CpG motifs contained in the minimal promoter region (−250 to −120 bp). Using the CpG-free pCpGL luciferase vector, we demonstrated that methylation of the DP1 promoter by a CpG methyltransferase (M. SssI) dramatically decreased the DP1 promoter activity, lending further support for the involvement of DNA methylation in the regulation of the DP1 promoter activity.

The effect of CpG methylation on the binding of Sp1 to its target sequences is controversial because of conflicting results. CpG methylation was reported to attenuate Sp1 binding to the promoter of GDF5 (Reynard et al., 2014), α-crystallin (CRYAA) (Liu et al., 2016), bromodomain-containing protein 7 (BRD7) (Liu et al., 2008), and organic cation-transporter 2 (OCT2) (Aoki et al., 2008). On the other hand, other studies reported that CpG methylation did not affect the binding activity of Sp1 to the promoter of claudin 4 (CLDN4) (Honda et al., 2006), luteinizing hormone receptor (*LHR*) (Zhang et al., 2005), p21 (Cip1) (Zhu et al., 2003), Toll-like receptor 2 (TLR2) (Furuta et al., 2008), and Protein Phosphatase 2Acα (Sunahori et al., 2009). In the present study, we demonstrated that CpG methylation of the Sp1 binding sites in the DP1 promoter has no effect on the binding activity of Sp1, suggesting that mechanisms other than inhibition of Sp1 binding are responsible for the DNA methylation-dependent regulation of DP1 transcription in chondrocytes (Figures 4, 5A). In addition to blocking the binding of transcription factors, DNA methylation can modulate transcription via a mechanism involving methyl binding proteins that bind to methylated DNA and recruit co-repressor molecules to silence gene expression (Ginder and Williams, 2018). Such a mechanism was observed at the CLD4 promoter (Honda et al., 2006). DNA methylation was shown to downregulate the expression of the CLDN4 gene without affecting the binding of Sp1, and this was associated with the recruitment of methyl-CpG-binding domain protein 2 (MBD2) (Honda et al., 2006). Further studies are needed to determine whether the recruitment of such proteins modulates DP1 expression in chondrocytes.

Previous studies have described the relation between DNA methylation and DP1 expression. DNA hypermethylation correlates with decreased DP1 expression in colorectal (Kalmar et al., 2015) and gastric (Kim et al., 2018) cancer, hypomethylation correlates with increased levels of DP1 expression in asthma (Isidoro-Garcia et al., 2011). Ambiguous results were described in colon cancer (Spisak et al., 2012) and neuroblastoma cell lines (Sugino et al., 2007).

Increasing evidence suggests that epigenetic changes may be involved in the pathogenesis of OA, and several genes involved in cartilage biology and OA pathogenesis were reported to be modulated by DNA methylation. The regulated genes include genes encoding ECM proteins such as type IX (Imagawa et al., 2014) and type X collagen (Zimmermann et al., 2008); cartilage degrading enzymes such as MMP-13 (Hashimoto et al., 2009) and ADAMTS-4 (Cheung et al., 2009); transcription factors such as RUNX2 (Takahashi et al., 2017; Rice et al., 2018), SOX9 (Kim et al., 2013) and PPARγ (Zhu et al., 2019); cytokines such as IL-1 (Hashimoto et al., 2009), leptin (Iliopoulos et al., 2007), and IL-8 (Takahashi et al., 2015); and growth factors such as BMP7 (Loeser et al., 2009), GDF5 (Reynard et al., 2014) and TGFβ1 (Rice et al., 2021). Interestingly, Zhu et al. reported that treatment of chondrocytes with the demethylating agent 5-Aza prevented IL-1-mediated downregulation of aggrecan, Col2, catalase, and superoxide dismutase 2 as well as IL-induced upregulation of MMP-13 and ADAMTS5, suggesting that 5-Aza has anti-OA properties. Indeed, treatment with 5-Aza was protective in a mouse model of instability-induced OA (Zhu et al., 2019).

Since we found that DNA methylation regulate DP1 expression in cultured chondrocytes, we analyzed the methylation status of the DP1 promoter region in genomic DNA isolated directly from normal and OA knee cartilage. There was no difference in the methylation status of the DP1 promoter between normal and OA cartilage at any CpG site analyzed. Epigenetic mechanisms other than DNA methylation may contribute to the regulation of DP1 expression in cartilage. Indeed, histone modifications were previously shown to participate in the regulation of DP1 expression (Sugino et al., 2007). It is noteworthy that long non-coding RNAs (lncRNAs) also known for their role in the regulation of gene expression were also suggested to regulate DP1 expression (Tang et al., 2019). Further studies are needed to determine whether such mechanisms contribute to the regulation of DP1 expression in cartilage.

We also compared the expression level of DP1 in OA and normal cartilage and found no difference, which is consistent with the similar methylation level of the DP1 proximal promoter in both groups. Several studies have examined the expression level of DP1 in healthy and diseased tissues. For instance, DP1 expression was shown to be upregulated on circulating basophils from patients with systemic lupus erythematosus (Pellefigues et al., 2018), in microglia and astrocytes within senile plaques from individuals with Alzheimer’s (Mohri, 2007), and in cells of the crypt epithelium of patients with ulcerative colitis (Vong et al., 2010). On the other hand, DP1 was shown to be downregulated in the adenoma-carcinoma sequence of patients with colorectal cancer (Kalmar et al., 2015), in intestinal metaplasia and gastric tumor cells from patients with intestinal-type early gastric cancer (Kim et al., 2018), and in artery smooth muscle cells from patients with idiopathic pulmonary arterial hypertension (He et al., 2020). Collectively, these data demonstrate the complexity of DP1 expression patterns in healthy and diseased tissues.

The comparable expression of DP1 in healthy and OA cartilage suggests that changes in DP1 levels do not contribute to the pathogenesis of OA. It is thus possible that the pathogenesis of OA is associated with a reduced level of the endogenous activator of DP1, PGD_2_. This is unlikely since we have previously demonstrated that the level of PGD_2_ is increased in OA synovial fluids (Zayed et al., 2008b). Another possibility is that OA is associated with alterations in the level of the downstream effectors of DP1. Indeed, the DP1 receptor signals through activation of the cAMP-PKA/CREB pathway (Crider et al., 1999; Hammad et al., 2007; Choi et al., 2011; Bassal et al., 2016), and previous studies reported that components of this pathway are dysregulated in OA cartilage (Zhang, 2022; Cai, 2023). These findings indicate that OA is associated with alterations in the down-stream effectors of DP1 and suggest that this may contributes, at least partially, to the development and or progression of the disease.

This study has some limitations. First, our analyses focused only on the role of Sp1 in the regulation of DP1 expression, and we cannot rule out the implication of other transcription factors not analyzed in the present study. Second, the sample size was small, which may have limited the statistical power to detect differences between control and OA cartilage. Third, we only measured the level of DP1 mRNA. We did not analyze the level of its protein level (Michel et al., 2009). Fourth, we analyzed CpG methylation using healthy non-OA cartilage and unaffected OA cartilage. This approach characterizes the methylation status of CpG motifs at the early stages of cartilage degeneration but cannot detect methylation changes at the latter stages of cartilage degeneration. Therefore, our future studies will compare CpG methylation levels of healthy non-OA cartilage with unaffected OA cartilage and with damaged OA cartilage. Fifth, we extracted RNA and DNA from full thickness cartilage samples including all layers, and it is known that gene expression in cartilage varies between its layers. Further studies of the role of DNA methylation in the pathogenesis of OA will have to include analysis of target gene expression and promoter methylation in different cartilage zones. Another limitation of our study is that we characterized only CpG motifs inside the DP1 promoter region spanning nucleotides −250 to −120, and we cannot exclude changes in the methylation status of other CpG residues not analyzed here. Further studies are needed to investigate the methylation status of CpG motifs located in other regions of the DP1 promoter, the 3′UTR and the gene body regions. Finally, OA cartilage tissues were obtained from subjects with end-stage OA, thus our findings may not reflect cartilage changes associated with the initiation and/or the progression of the disease.

In summary, we have characterized the human DP1 gene promoter and showed a significant role for Sp1 in the regulation of its activity. We have also shown that transcription of DP1 may be regulated by CpG methylation. Additionally, we found that the expression level of DP1 and the methylation status of its promoter were not different between OA cartilage and normal tissues. Further studies are necessary to evaluate the role of other epigenetic mechanisms in the regulation of DP1 gene expression.

## Data Availability

The raw data supporting the conclusion of this article will be made available by the authors, without undue reservation.
